# A predictive model for low-dose rituximab response in anti-acetylcholine receptor antibody-positive myasthenia gravis: establishment and validation

**DOI:** 10.3389/fimmu.2026.1781372

**Published:** 2026-06-03

**Authors:** Chenlu Hou, Yifan Zhang, Xiangqi Cao, Yonglan Tang, Ting Gao, Baoli Tang, Ying Zhu, Zhe Ruan, Ting Chang

**Affiliations:** 1 Department of Neurology, Tangdu Hospital, The Fourth Military Medical University, Xi’an, China; 2 School of Medicine, Northwest University, Xi’an, China

**Keywords:** B cells, myasthenia gravis, precision medicine, prediction model, rituximab

## Abstract

**Background:**

Rituximab (RTX) represents an established therapeutic option for myasthenia gravis refractory to conventional immunotherapy. However, its heterogeneous efficacy across populations creates a challenge in clinical application, necessitating the development of robust predictive models to better identify patients likely to derive meaningful benefit from this treatment.

**Methods:**

This study retrospectively reviewed the patients who visited the research center and received RTX treatment between August 2019 and January 2025. The primary outcome measure was minimal symptom expression (MSE). A predictive model was constructed using logistic regression and presented in a nomogram. The performance of the model was evaluated by calculating the area under the receiver operating characteristic curve (AUC), with internal validation performed via the bootstrap method. The study population was divided into high- and low- probability groups according to Youden index. Calibration curves with 1000 replications bootstrap resampling were plotted to visualize the calibration of the nomogram. Decision curve analyzes (DCA) with 1000 replications bootstrap resampling were per formed to evaluate the clinical usefulness of the model.

**Results:**

A total of 117 patients were included. Five variables were screened including new-onset MG, baseline MG-ADL score, high CD19^+^/CD27^+^ B lymphocyte proportion, high-dose prednisone, and early immunotherapy initiation. The model exhibited moderate discriminative ability with AUC value 0.777. Using a threshold probability of 0.534 derived from the Youden index, patients were stratified into high- and low-probability groups (sensitivity 72.7%, specificity 71.0%). The high-probability group showed a 6.52-fold higher likelihood of achieving MSE. The calibration plot showed that when the probability was between 0 and 0.7, the deviation calibration curve of the model was consistent with the ideal curve.

**Conclusion:**

This nomogram helps identify AChR-MG patients likely to attain MSE 6 months after low-dose rituximab, thereby supporting personalized therapeutic decision-making.

## Introduction

Myasthenia gravis (MG) is an autoimmune disorder mediated by antibodies and characterized by dysfunctional neuromuscular junction (NMJ) transmission. In most cases, the pathogenic antibody targets the acetylcholine receptor (AChR-Ab) on the postsynaptic membrane of the NMJ (1).

B cells exert diverse immunoregulatory effects through cytokines that influence T cell activation, plasma cell differentiation, and complement activation, thereby playing a pivotal role in the pathogenesis and progression of MG (2). Given this role, targeting B cells has emerged as a key therapeutic strategy for MG. Rituximab (RTX), a human-mouse chimeric anti-CD20 monoclonal antibody, acts by specifically binding to CD20 molecules expressed on the surface of pre-B cells and mature B cells. The agent primarily exerts its effects through antibody-dependent cellular cytotoxicity (ADCC) and complement-dependent cytotoxicity (CDC), thereby producing B-cell depletion and delivering therapeutic benefit in B cell-mediated autoimmune disorders such as MG (3). Extensive clinical evidence supports the efficacy of low-dose RTX in MG. Notably, a 2019 systematic review reported a 68% response rate in AChR-MG (4). The RINOMAX study showed that 71% of new-onset generalized MG patients achieved minimal manifestations (MMS) at week 16 following a single 500 mg dose of RTX (MMS: Quantitative MG Score ≤4, prednisone ≤10 mg/d), a rate substantially higher than that observed in the placebo group (5). Therefore, RTX (a B cell-depleting agent) remains a valuable therapeutic option for MG patients who are unresponsive to or intolerant of conventional immunotherapy.

However, RTX treatment in MG still faces notable risks and challenges. First, despite years of clinical application, the efficacy remains debated, with reported response rates varying considerably across studies, ranging from 30% to 70%. Meta-analyzes further indicated that approximately 44% to 70% of patients achieved the MMS or better, as defined by the Myasthenia Gravis Foundation of America Post-Intervention Status (MGFA-PIS), following RTX treatment (6, 7). However, in the BeatMG study, 60% of AChR-MG patients receiving two RTX cycles attained the primary steroid-sparing outcome, which did not differ significantly from the 56% rate observed in the placebo group (8). Collectively, these results indicate that RTX still carries a risk of failure, highlighting the need for of careful patient stratification to optimize its clinical efficacy. Second, as a B cell-depleting therapy (BCDT), RTX is associated with a range of adverse events such as infusion reactions, heart failure, and sustained B cell depletion (9). Long-term peripheral B lymphocyte depletion significantly raises the risk of infections, posing both life-threatening risks in severe instances and considerable treatment burdens for patients. A large-scale retrospective cohort study reported that 11% of patients developed mild, transient infusion reactions to RTX. Notably, 3% of patients experienced severe allergic reactions with recurrent pneumonia, leading to treatment discontinuation (10). Additionally, controversy persists regarding the selection of MG patients who may benefit from RTX, and predictors of its efficacy require further investigation. Clarifying factors influencing RTX response, improving patient selection, and reducing treatment risks are urgent priorities. Thus, establishing a predictive model for RTX responsiveness is crucial to optimizing its targeted use in MG therapy.

In summary, this study aims to establish and validate a prediction model based on treatment response data from AChR-MG patients receiving low-dose rituximab. The model will predict the probability of achieving MSE at 6 months post-RTX, screen RTX-beneficial MG patients, and ultimately provide a basis for targeted RTX application in MG.

## Materials and methods

### Study design

This study is a single-center retrospective cohort study. The research data were derived from the MG Registration Cohort of Tangdu Hospital of Air Force Medical University (Registration Number: ChiCTR2100043273). This study included data of MG patients who received RTX treatment between August 2019 and January 2025, as recorded in the database. Patients registered in this database typically undergo regular follow-up visits, with an interval of 1 to 3 months between visits. The data in this database have been previously used in a number of studies (11–13).

### Study population

The inclusion criteria of this study were as follows: (1) Age ≥ 18 years; (2) Diagnosis of MG by the neurologist; (3) Positive for AChR-Ab; (4) Myasthenia Gravis-Activities of Daily Living (MG-ADL) score ≥ 2 points; (5) Receipt of 500 mg RTX treatment; (6) The time from the first administration of RTX to the last follow-up was at least 6 months.

Patients with any of the following criteria were excluded: (1) With incomplete key follow-up data; (2) With concurrent other neurological disorders that might interfere with the assessment of MG status; (3) With comorbidities requiring additional immunosuppressive therapy. Data including demographic characteristics of patients, baseline disease-related data, medication treatment data, and follow-up data were collected.

### Baseline characteristics and potential predictive variables

Baseline variables were extracted from the database and verified by reviewing electronic medical records. These variables include: (1) Demographic characteristics: age, onset age, and gender; (2) Baseline clinical characteristics: Myasthenia Gravis Foundation of America (MGFA) classification, early-onset myasthenia gravis (EOMG), late-onset myasthenia gravis (LOMG), disease duration, duration of initiating immunotherapy, duration of immunotherapy, presence of other MG-related antibodies, history of thymectomy, thymectomy duration, new-onset MG, refractory MG, baseline MG-ADL score, peripheral blood B lymphocyte subsets, daily dose of cholinesterase inhibitors, daily dose of prednisone, previous immunosuppressive therapy, history of relapse and exacerbation, history of rescue therapy, and history of myasthenic crisis.

EOMG was defined as onset age < 50 years, and LOMG as onset age ≥ 50 years. Disease duration was the time from symptom onset to the first RTX administration. The time from disease onset to initial immunosuppressive therapy was defined as the duration of initiating immunotherapy, while the duration of immunotherapy referred to the interval from first immunosuppressive treatment to RTX administration. New-onset MG was defined as disease where the interval from symptom onset to first RTX dose was ≤ 12 months. Refractory MG was defined as inadequate disease control despite standardized first-line and second-line therapies, characterized by persistent prominent symptoms or recurrent exacerbations that significantly impair quality of life (10).

### Outcome measure

The primary outcome measure was defined as the achievement of MSE in AChR-MG patients at 6 months after treatment with low-dose RTX, which was specified as the MG-ADL score ≤ 1 point (14).

Data from the database were supplemented through medical record review, and patients with missing key variables were excluded. All continuous variables were tested for normality using the Shapiro-Wilk test. Normally distributed continuous variables were presented as mean ± standard deviation, while non-normally distributed data were expressed as median (interquartile range, IQR). Categorical variables were reported as frequency (percentages). Between-cohort comparisons of categorical variables were performed using the chi-square test or Fisher’s exact test, and continuous variables were compared via the Student’s t-test (for normal distribution) or Mann-Whitney U test (for non-normal distribution).

A logistic regression model was constructed for prediction. Prior to inclusion in the final model, variables were categorized based on relevant literature and the maximum Youden index, and multicollinearity among variables was assessed using the variance inflation factor (VIF). Based on multivariate logistic regression analysis, a nomogram was generated via the “rms” package in R software to facilitate the clinical application of the prediction model. To evaluate model performance, receiver operating characteristic (ROC) curve analysis was performed, and the AUC was calculated. Additionally, a bootstrap resampling method with 1000 repetitions was used to estimate the 95% confidence interval (CI) of the AUC (15). The cut-off value of the model was determined according to the maximum Youden index (Youden index = sensitivity + specificity - 1) (16). A calibration curve was plotted via bootstrap resampling with 1000 repetitions to visually demonstrate the calibration effect of the nomogram. Additionally, the Hosmer-Lemeshow chi-square test was applied to assess the goodness-of-fit of the model, verifying whether the model as a whole could well fit the actual data. To validate the clinical utility of the model, we plotted a DCA graph with 1000 repeated bootstrap resamplings using the “rmda” package in R software. This graph clearly shows the potential net benefit of the model under different threshold probabilities (17). Finally, according to the cut-off probability calculated by the Youden index, the study population was divided into a high-probability group and a low-probability group. Multivariate logistic regression analysis was used to compare the risk difference in achieving MSE between the two groups of patients after 6 months of low-dose RTX treatment. Internal validation of the model was conducted using the bootstrap method with 1000 repetitions to calculate the mean AUC value.

The data analysis in this study was performed using IBM SPSS Version 24.0 and R Software Version 4.2.0 (http://www.Rproject.org). Statistical significance was defined as a two-tailed *p* < 0.05.

## Results

### General characteristics of the study population

**Figure 1:**
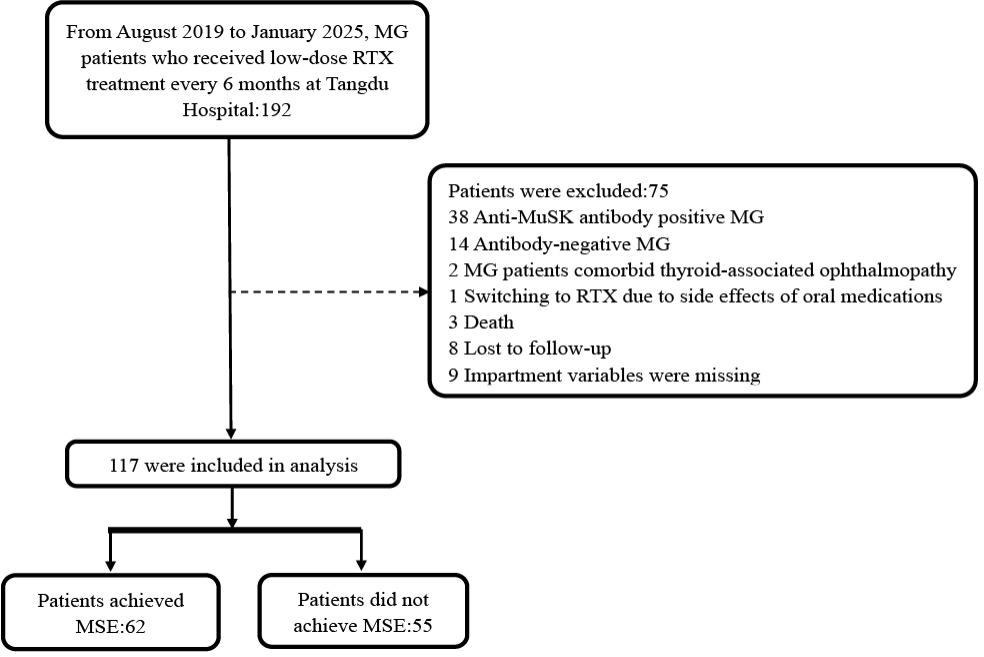
Study flowchart. Flow diagram describing patient selection.

A total of 192 MG patients from the database met the inclusion criteria, and 75 patients were excluded based on the exclusion criteria. Of the patients who met the inclusion criteria, 9 were excluded owing to missing candidate variables. The missing ratio was 7%. Finally, 117 AChR-MG patients were included in the study. The screening process is shown in **Figure 1**.

Of the 117 patients who met the inclusion criteria, there were 54 males and 63 females. The median age was 45 years (range: 18.00-79.00 years), the median age of males was 53 years (range: 37.00-60.00 years) while the median age of females was 36 years (range: 24.75-56.00 years). The median age at onset was 43 years (range: 5.00-79.00 years). A total of 62 patients achieved MSE at 6 months after receiving RTX treatment (52.99%), while 55 patients did not achieve MSE (47.01%). Comparisons between the two groups revealed several significant differences. The proportion of patients with refractory MG was lower in the MSE group than in the non-MSE group (29.03% *vs*. 47.27%, p = 0.042). The MG-ADL score was lower in the MSE group (5.45 ± 2.84 *vs*. 7.07 ± 3.55, p = 0.008). The daily dose of cholinesterase inhibitors was lower in the MSE group than in the non-MSE group (180 ± 180, 270 *vs*. 255 ± 180, 360, p = 0.008). The proportion of patients with a history of combined immunosuppressive therapy was lower in the MSE group than in the non-MSE group (43.55% *vs*. 63.64%, *p* = 0.030). No significant differences were discovered in other baseline characteristics between the MSE group and the non-MSE group. Detailed data are presented in **Table 1**.

**Table 1:** Baseline demographic characteristics of MSE and non-MSE(n=117).

Baseline characteristics	MSE (n=62)	Non-MSE (n=55)	P
Sex, n (%)			0.819
Male	28.00 (45.16)	26.00 (47.27)	
Female	34.00 (54.84)	29.00 (52.73)	
Age, Median (Q1, Q3), years	48.50 (31.50,60.00)	42.00 (28.00,59.00)	0.619
MGFA classification, n (%)			0.256
I	17.00 (27.42)	13.00 (23.64)	
II	32.00 (51.61)	23.00 (41.82)	
≥III	13.00 (20.97)	19.00 (34.55)	
Onset age, Median (Q1, Q3)	45.50 (27.50,57.25)	41.00 (24.00,55.00)	0.592
EOMG, n (%)	37.00 (59.68)	35.00 (63.64)	0.660
LOMG, n (%)	25.00 (40.32)	20.00 (36.36)	0.706
Disease duration, Median (Q1, Q3), months	26.50 (6.00,70.00)	21.00 (12.00,52.00)	0.766
Duration of initiating immunotherapy, Median (Q1, Q3), months	1.00 (0.00,8.75)	2.00 (0.00,5.00)	0.847
Duration of immunotherapy, Median (Q1, Q3), months	14.50 (1.75,60.25)	14.00 (9.00,51.00)	0.205
Comorbid other MG related antibodies, n (%)	24.00 (38.71)	22.00 (40.00)	0.887
Thymic status, n (%)			0.617
Normal	40.00 (64.52)	40.00 (72.73)	
Thymoma	15.00 (24.19)	10.00 (18.18)	
Thymic hyperplasia	7.00 (11.29)	6.00 (10.91)	
Thymectomy, n (%)	16.00 (25.81)	11.00 (20.00)	0.457
Thymectomy duration, Median (Q1, Q3), months	3.00 (2.00,6.00)	6.50(1.50,11.00)	0.492
New-onset MG, n (%)	22.00 (35.48)	14.00 (25.45)	0.241
Refractory MG, n (%)	18.00 (29.03)	26.00 (47.27)	0.042*
Baseline MG-ADL, Mean (SD), years	5.45 (2.84)	7.07 (3.55)	0.008*
Peripheral blood B cell subsets			
Proportion of CD19^+^ B lymphocytes, Median (Q1, Q3), %	10.14 (6.24,16.11)	12.13 (8.21,15.24)	0.335
Proportion of CD19^+^/CD27^+^ B lymphocytes, Median (Q1, Q3), %	2.80 (1.94,5.16)	3.93 (2.37,5.54)	0.105
Proportion of CD20^+^ B lymphocytes, Median (Q1, Q3), %	10.00 (6.09,15.70)	12.00 (8.65,15.2)	0.241
Cholinesterase inhibitor dosage, Median (Q1, Q3), mg/d	180.00 (180.00,270.00)	255.00 (180.00,360.00)	0.008*
Prednisone dosage, Median (Q1, Q3), mg/d	15.00 (0.00,20.00)	15.00 (0.00,20.00)	0.188
Previous combined immunosuppression, n (%)	27.00 (43.55)	35.00 (63.64)	0.030*
AZA	6.00 (9.68)	4.00 (7.27)	
TAC	16.00 (25.81)	20.00 (36.36)	
MMF	4.00 (6.45)	6.00 (10.91)	
Efgartigimod	6.00 (9.68)	3.00 (5.45)	
Eculizumab	4.00 (6.45)	1.00 (1.82)	
Tocilizumab	2.00 (3.23)	6.00 (10.91)	
History of recurrence or exacerbation, n (%)	54.00 (87.10)	47.00 (85.45)	0.999
History of rescue therapy, n (%)	21.00 (33.87)	20.00 (36.36)	0.778
History of MC, n (%)	4.00 (6.45)	6.00 (10.91)	0.743

*indicates that P < 0.05.

MGFA, Myasthenia Gravis Foundation of America; EOMG, Early-Onset Myasthenia gravis; LOMG, Late-Onset Myasthenia gravis; MG-ADL, MG-related activities of daily living; AZA, azathioprine; TAC, Tacrolimus; MMF, Mycophenolate mofetil; MC, Myasthenic crisis.

### Development of the model

The optimal model was selected using the backward regression method, following univariate logistic regression screening (**Supplementary Table 1**) and subsequent multivariate logistic regression validation. Five clinical features were finally identified as candidate variables for predicting the efficacy of low-dose RTX treatment after 6 months. These variables included early initiation of immunosuppressive therapy, new-onset MG, MG-ADL score, high proportion of CD19^+^/CD27^+^ B lymphocytes, and high dose of prednisone. The duration of immunosuppressive therapy was divided into an early initiation group and a late initiation group based on whether immunosuppressive therapy was administered within 12 months of disease onset. Using the maximum Youden index, the threshold for CD19^+^/CD27^+^ B lymphocytes proportion was determined to be 2.515%. Patients were then classified into a high- and a low-proportion groups based on the threshold. Cholinesterase inhibitors and prednisone were each divided into a high-dose group and a low-dose group according to their daily doses (i.e., daily dose of pyridostigmine ≥ 180 mg, daily dose of prednisone ≥ 40 mg) (**Supplementary Table 2**). The simplified models were compared with the full model by the likelihood ratio test, and the goodness of fit revealed no significant differences (p >0.05). The variance inflation factor (VIF) test indicated that there was no multicollinearity among all included variables (VIF values of all variables were<10). Multivariate logistic regression analysis showed that new-onset MG (odds ratio [OR] = 3.446; 95% confidence interval [95% CI]: 1.280–9.280; p = 0.014) was a potential protective factor for achieving MSE after 6 months of low-dose RTX treatment (**Table 2****).**

**Table 2:** Adjusted OR of the multivariable model.

Predictive variables	OR	95% CI	P
new-onset MG	3.45	1.28-9.28	<0.01
Baseline MG-ADL	0.82	0.71-0.94	0.01
high proportion of CD19^+^/CD27^+^ B lymphocytes	0.33	0.13-0.81	0.02
high dose prednisone	0.28	0.08-1.03	0.05
early initiation of immunotherapy	0.13	0.03-0.53	<0.01

CI, confidence interval; MG-ADL, MG-related activities of daily living; high proportion of CD19^+^/CD27^+^ B lymphocytes, the proportion of CD19^+^/CD27^+^ B lymphocytes≥2.515%; high dose prednisone, the dose of prednisone≥40mg/d; early initiation of immunotherapy, the duration from onset to initiation of immunotherapy≤12 months.

### Presentation of the model

**Figure 2:**
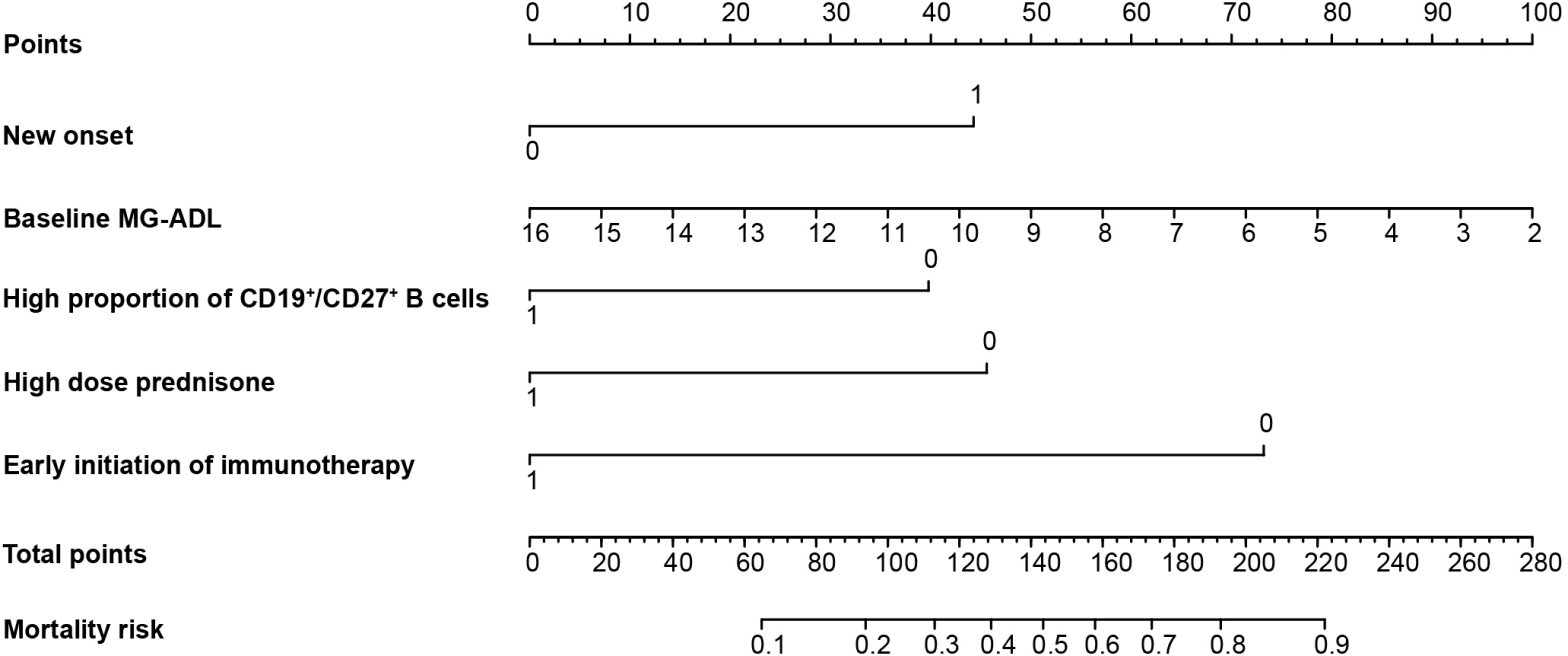
Nomogram predicting the probability of MSE. The nomogram summed the points identified on the scale for each variable. The total points projected on the bottom scales indicate the MSE probabilities.

The nomogram for predicting the probability of achieving MSE in AChR-MG patients after 6 months of low-dose RTX treatment is shown in **Figure 2**. This nomogram converts the regression coefficients of the 5 predictive variables calculated in the multivariate logistic regression analysis into scores ranging from 0 to 100. The total score is obtained by summing the scores corresponding to each predictive variable of a patient; then, by locating this total score on the total score scale, the individual probability of the patient achieving the MSE status after 6 months of low-dose RTX treatment can be determined (18).

### Validation of the model

**Figure 3:**
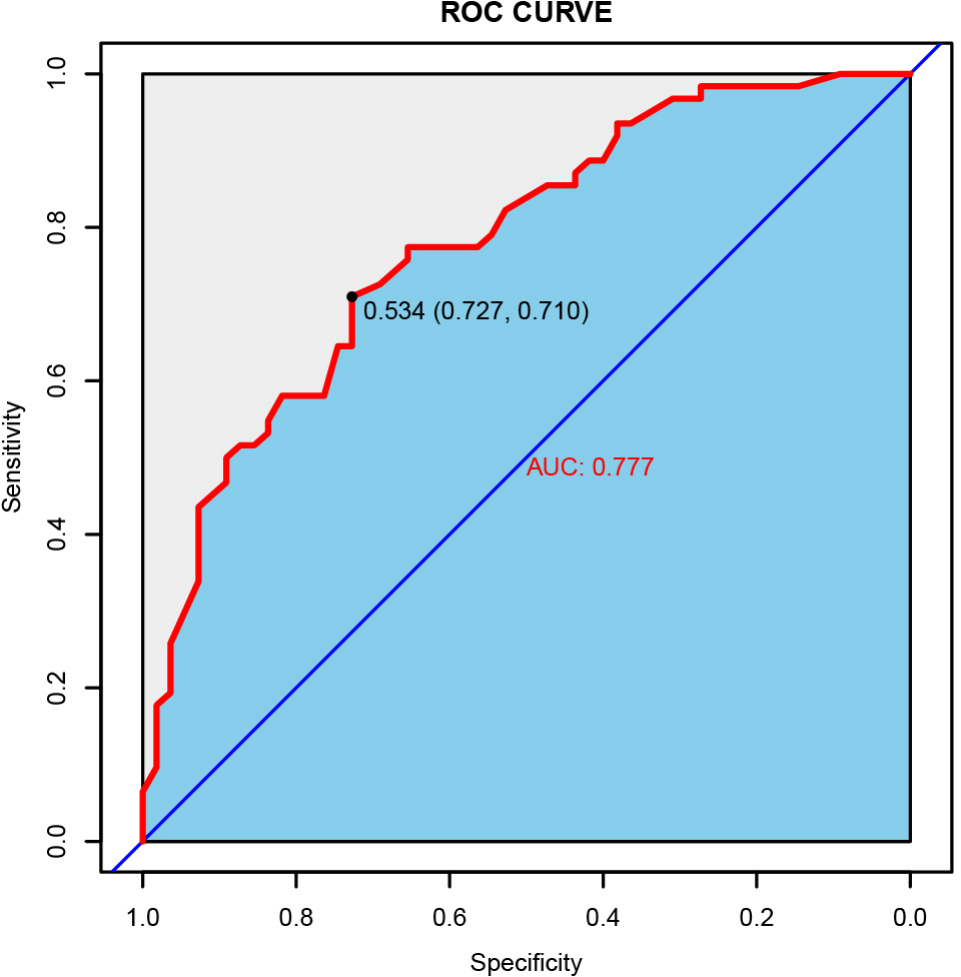
The receiver operating characteristic (ROC) curve of the predictive model to predict MSE. The area under the curve was 0.777 (95% CI: 0.694–0.861). The 95% interval of specificity calculated by bootstrap.

The discriminative ability of the model was evaluated by plotting the ROC curve and calculating the AUC. The validated AUC value was 0.777 (95% CI: 0.694–0.861). The threshold probability calculated via the Youden index was 0.534, which was used to distinguish the high-probability group and the low-probability group (**Figure 3**). Internal validation of the model was performed using a bootstrap method with 1000 repetitions to reduce the risk of overfitting, and the validated mean AUC value was 0.746. When the threshold was 0.534, the model had a sensitivity of 72.7% and a specificity of 71.0%. Compared to the low-probability group, patients in the high-probability group had a 6.519-fold higher probability of achieving MSE after 6 months of low-dose RTX treatment (odds ratio [OR] = 6.519; 95% CI: 2.906–14.624; p < 0.001) (**Table 3**).

**Table 3:** Compared with the low-probability group, the risk of MSE in the high-probability group.

Groups	OR	95% CI	P
the low-probability group			
the high-probability group	6.52	2.91-14.62	p<0.01

CI, confidence interval; OR, odds ratio; MSE, minimal symptom expression.

**Figure 4:**
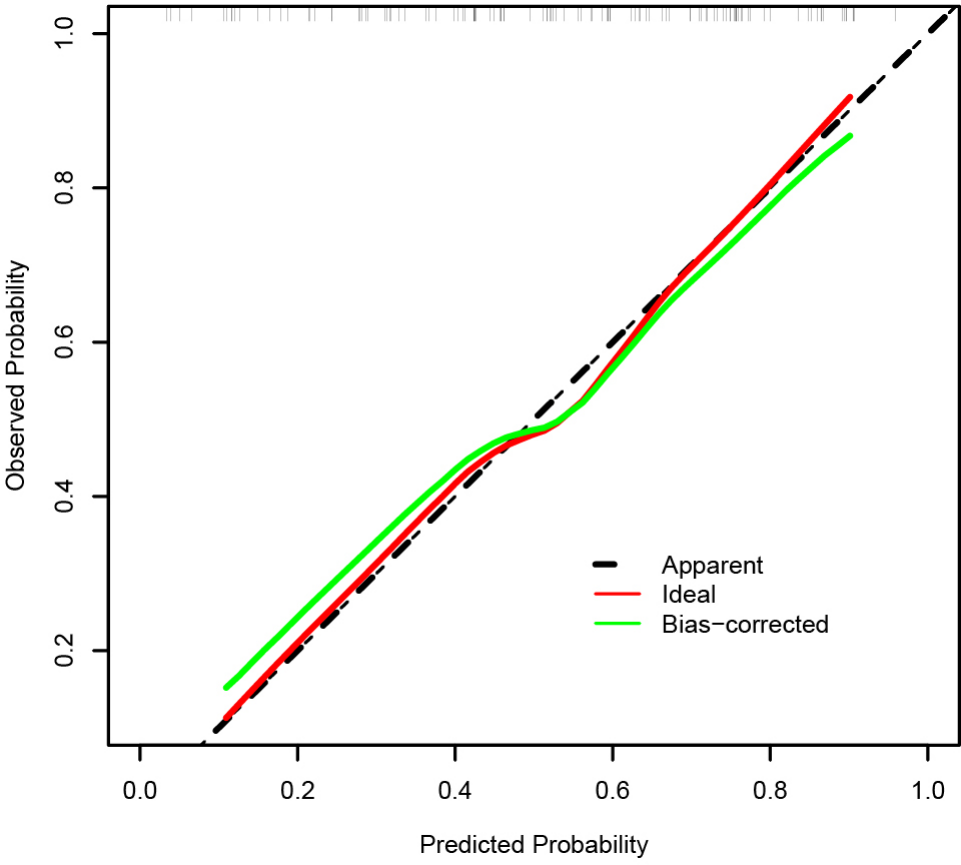
Calibration curve of the nomogram for predicting MSE at 6 months after a single 500 mg dose of RTX in patients with AChR-MG. The dashed diagonal line represents the ideal calibration line. The red solid curve represents the bias-corrected calibration curve, and the green solid curve represents the apparent calibration curve. The red bias-corrected curve closely follows the ideal line across most probability ranges, indicating good overall calibration.

The calibration plot indicated that when the predicted probability ranged from 0.18 to 0.88, the bias-corrected curve was generally consistent with the ideal curve. However, when the probability was in the range of 0.08–0.18, the model tended to underestimate the likelihood that AChR-MG patients would achieve MSE 6 months after receiving a single 500 mg dose of RTX. Conversely, when the predicted probability was in the range of 0.78–0.98, the model showed a tendency to overestimate the probability of achieving MSE (**Figure 4**).

**Figure 5:**
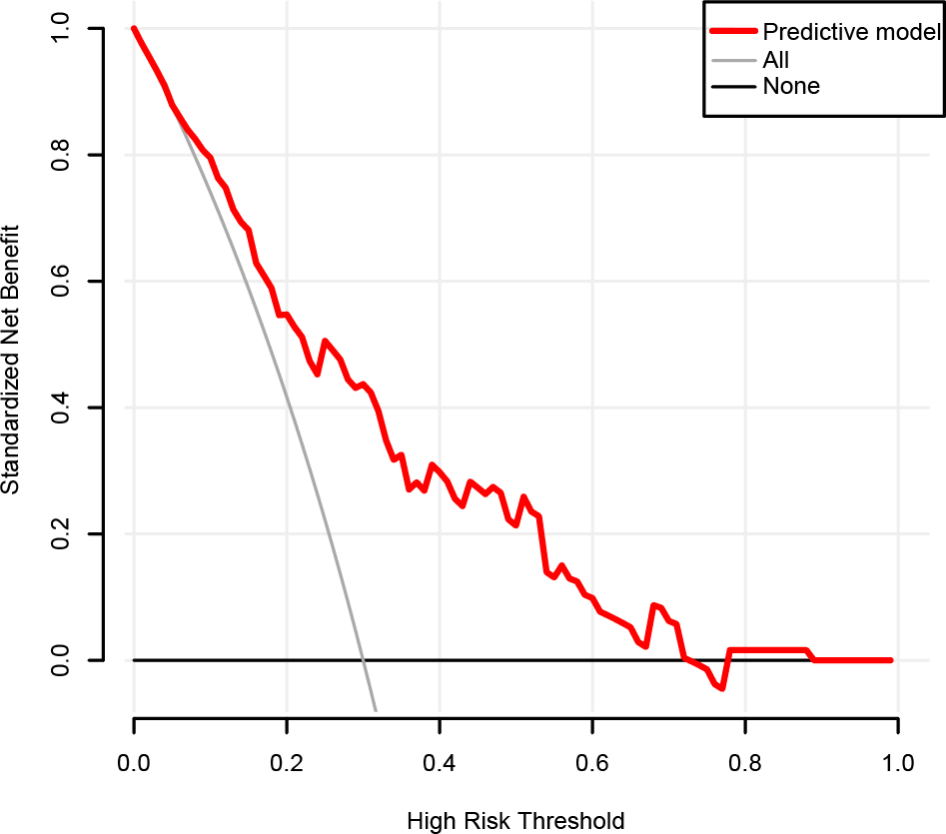
The decision curve analyzes (DCA) for the clinical values of this model. The Y-axis represents the net benefit, and the X-axis represents the MSE possibility. The gray line indicates the net benefit that all patients are considered not achieving MSE and treated. The black line indicates that all patients will not achieve MSE, and the net benefit is 0. The red curve indicates the net benefit at different threshold probabilities.

The results of decision curve analysis (DCA) related to the clinical utility of the model are shown in **Figure 5**. The x-axis represents the threshold probability of AChR-MG patients achieving MSE at 6 months after low-dose RTX treatment, and the y-axis represents the standard net benefit of using this model. Decision curve analysis reflects clinical utility through the area between model curves. For example, at a threshold of 0.53, the standardized net benefit of the model is approximately 0.3, showing good net benefit ability and certain value in aiding clinical decision-making.

## Discussion

Based on a MG registry cohort, this study developed and validated a predictive model for identifying AChR-MG patients who achieve MSE after 6 months of low-dose rituximab treatment. The model was presented in the form of a nomogram. We found that high score of MG-ADL, high proportion of CD19^+^/CD27^+^B lymphocytes, high dose prednisone and early initiation of immunotherapy were potential risk factors for MSE. This model had moderate predictive ability and could help clinicians to a certain extent accurately select MG patients who are suitable for RTX treatment.

Currently available prediction models for RTX treatment include the one proposed by Zhou et al. (2024) (13). Their study constructed a predictive model for short-term efficacy of low-dose rituximab in patients with MG They identified that shorter disease duration, positive anti-muscle-specific tyrosine kinase (MuSK) antibodies, and the AA genotype at the rs1801274 locus of the FCGR2A gene were significant predictive factors for achieving clinical response within 6 months in MG patients treated with 600 mg RTX. However, this study included genotype as a final predictive factor, the high cost of genetic testing may limit its clinical application. Additionally, due to the limitation of sample size the statistical power of its multivariate logistic regression analysis may be insufficient.

A prospective study found that compared with patients in the refractory group, patients with new-onset generalized myasthenia gravis (gMG) had better clinical outcomes after receiving RTX treatment. This result is basically consistent with the conclusion of this study, namely that new-onset MG is a favorable predictive factor for the clinical response to RTX treatment in AChR-MG (10). Consistently, in the RINOMAX randomized clinical trial, MG patients who developed generalized symptoms within 12 months of disease onset had a higher probability of achieving minimal manifestations of MG after receiving a single 500 mg dose of RTX (5). From a pathophysiological perspective, B cells attack the neuromuscular junction by producing autoantibodies, leading to muscle weakness and fatigue (19). Early intervention can inhibit the production of abnormal antibodies in a timelier manner. RTX targets B cells expressing the CD20 antigen, leading to their depletion and thus reducing the production of autoantibodies (20, 21). Therefore, timely initiation of RTX treatment in the early stage of MG onset seems to be more conducive to achieving a clinical response.

In addition, we found that a high proportion of CD19^+^/CD27^+^ B lymphocytes before RTX treatment is a potential risk factor for achieving MSE at 6 months post-treatment in patients with AChR-MG. This is consistent with the results of a multicenter study conducted in patients with rheumatoid arthritis (22), which showed that a low proportion of baseline memory B cells is associated with a better therapeutic response to RTX. However, no relevant studies have been conducted in the field of MG. The mechanism of action of RTX involves specifically binding to the CD20 molecule on the surface of B cells to deplete mature B cells in the peripheral circulation, but it has extremely weak ability to deplete memory B cells that do not express or weakly express CD20, especially long-lived memory B cells (2, 22). When the baseline count of memory B cells in patients is too high, RTX cannot effectively deplete these cells; instead, they persist as an “autoantibody-producing reservoir,” thereby impairing the therapeutic efficacy of RTX (23). Secondly, the residual memory B cells can rapidly differentiate into plasma cells and re-produce pathogenic antibodies such as anti-AChR antibodies, leading to insignificant symptom relief after treatment or disease recurrence within a short period (i.e., several months to one year) (8). To date, only one study has confirmed that when the proportion of memory B cells exceeds 0.05%, repeated RTX infusions may help control the disease and reduce the recurrence rate (24). However, further research is warranted to elucidate the mechanistic role of memory B cells in RTX treatment for MG, as well as to define the treatment-related threshold values. Whether the dosage of RTX should be increased for patients with a high proportion of baseline memory B cells also requires further investigation.

Furthermore, we observed that in patients with MG, the use of high-dose prednisone prior to RTX treatment may diminish the efficacy of RTX. Currently, prednisone remains a first-line therapy for MG, leading to symptomatic improvement in 70%–80% of patients (1). However, the administration of high-dose glucocorticoids before RTX treatment may impair its effectiveness through the following mechanisms: First, the continued requirement for high-dose prednisone prior to initiating second-line RTX therapy suggests that the patient has had a suboptimal or inadequate response to first-line treatments, indicating a more refractory condition. Second, the efficiency of B-cell depletion is critical to the therapeutic effect of RTX (25). Glucocorticoids exert potent immunosuppressive effects and can induce apoptosis of lymphocytes, including B cells (26). The use of high-dose glucocorticoids before RTX administration may lead to clonal replacement—where the original dominant clones are substantially reduced and replaced by more naïve B-cell lineages. As a result, RTX may fail to adequately bind to these newly generated B cells and initiate subsequent ADCC or CDC, thereby compromising its therapeutic efficacy (27). Additionally, high-dose glucocorticoids act not only on B cells but also influence T cells, macrophages, and other immune cells, while altering the cytokine network (28). This broad immunosuppressive effect may interfere with the specific B-cell depletion process mediated by RTX, thereby undermining its therapeutic role. Therefore, we propose that the use of high-dose glucocorticoids prior to RTX treatment may be a risk factor for achieving MSE in AChR-MG patients.

The suboptimal efficacy of RTX in MG patients who initiate immunosuppressive therapy early in the disease course is a multifaceted issue, involving factors such as drug mechanisms, B-cell depletion, disease heterogeneity, and timing of treatment. Early initiation of immunosuppressive therapy indicates that patients have previously received prednisone and/or non-hormonal immunosuppressants with inadequate response before transitioning to RTX as a second-line treatment. However, early use of immunosuppressants may alter the quantity and function of B cells, thereby compromising the targeting efficiency of RTX (29). For instance, mycophenolate mofetil, a non-hormonal immunosuppressant, has an active metabolite—mycophenolic acid (MPA)—that inhibits the Akt/mTOR and STAT5 pathways and induces reversible cellular quiescence in both T and B cells (30). After early exposure to such conventional immunosuppressants, the B-cell compartment may already be partially suppressed or altered. In particular, short-lived effector B cells and long-lived autoantibody-secreting plasma cells may be diminished or phenotypically modified by these treatments. This pre-existing immunosuppressed state may lead to less effective targeting and depletion of B cells by subsequent RTX administration compared with patients without such pretreatment. Moreover, the nonspecific immunomodulatory effects of conventional immunosuppressants may interfere with the precise mechanism of action of RTX. Therefore, AChR-MG patients who start treatment with Steroid therapy or other immunosuppression in the early stage of the disease are less likely to achieve MSE.

This study has several limitations: First, the retrospective design of this study may introduce potential biases. To mitigate this, we enrolled a relatively large cohort to develop the nomogram and performed internal validation to reduce bias inherent in retrospective data analysis. Additionally, incomplete medical records and unavoidable missing data led to the exclusion of certain variables (such as the Quantitative Myasthenia Gravis Score, QMGs), which may affect the predictive accuracy of the nomogram. We supplemented missing information as thoroughly as possible through medical record reviews and telephone follow-ups to minimize information bias. Second, variations in B-cell subset testing techniques across different centers may limit the generalizability of this nomogram to other patient populations. Moreover, the model currently lacks external validation, which will be addressed in future studies. Third, the limited number of outcome events and suboptimal discriminative ability of the model may lead to prediction inaccuracies. Clinicians should therefore integrate the model’s predictions with comprehensive clinical assessment when making decisions.

In conclusion, based on baseline clinical characteristics, this study developed and validated a model for predicting the probability of clinical response to rituximab. This model has potential value in guiding clinical decision-making for AChR-MG patients receiving low-dose rituximab treatment, and external validation is still needed in the future.

## Data Availability

The data analyzed in the study are available from the corresponding authors upon reasonable request.
